# Insulin-signalling dysregulation and inflammation is programmed trans-generationally in a female rat model of poor maternal nutrition

**DOI:** 10.1038/s41598-018-22383-w

**Published:** 2018-03-05

**Authors:** Jane L. Tarry-Adkins, Catherine E. Aiken, Thomas J. Ashmore, Susan E. Ozanne

**Affiliations:** University of Cambridge Metabolic Research Laboratories and MRC Metabolic Diseases Unit, Wellcome Trust-MRC Institute of Metabolic Science, Level 4, Box 289, Addenbrookes’ Treatment Centre, Addenbrookes’ Hospital, Hills Road, Cambridge, CB2 OQQ UK

## Abstract

Developmental programming phenotypes can be recapitulated in subsequent generations not directly exposed to the initial suboptimal intrauterine environment. A maternal low-protein diet during pregnancy and postnatal catch-up growth (‘recuperated’) alters insulin signaling and inflammation in rat offspring (F1-generation). We aimed to establish if this phenotype is also present in F2-generation females. Insulin-receptor-substrate-1 protein expression was decreased in para-ovarian adipose tissue at 3 months in offspring exposed to a grand-maternal low-protein diet (F2-recuperated), vs. F2-control animals (p < 0.05). There was no effect of grand-maternal diet upon *Insulin-receptor-substrate-1* mRNA. Protein-kinase C-zeta protein levels were increased at 3 and 6 months in F2-recuperated animals (p < 0.01 at both ages). Phosphorylated-Akt^ser473^ levels were decreased in F2-recuperated animals (p < 0.001). Interleukin-1β protein levels were increased at 3 (p < 0.01) and (p < 0.001) 6 months in F2-recuperated animals. Vastus-lateralis insulin-receptor-β protein expression (p < 0.001) and pAkt^ser473^ (p < 0.01) were increased at 3 months in F2-recuperated animals compared to controls. At 6 months, PAkt^ser473^ was lower in F2-recuperated animals (p < 0.001). Aspects of insulin signalling dysregulation and inflammation present in offspring of low-protein fed dams can be transmitted to subsequent generations without further exposure to a suboptimal maternal diet. These findings contribute to our understanding of insulin-resistance in grandchildren of sub-optimally nourished individuals during pregnancy.

## Introduction

A sub-optimal intrauterine environment leading to low birth weight followed by accelerated postnatal catch-up growth is associated with increased risk of later metabolic dysfunction in humans, including glucose intolerance^1^, insulin resistance^2,3^ and type 2 diabetes (T2D)^4–6^. This phenomenon (widely known as ‘developmental programming’) has also been recapitulated in animal models^7,8^. Interestingly, all these ‘programmable’ conditions have a common pro-inflammatory phenotype.

Insulin resistance, glucose intolerance, and incidence of developing type-2 diabetes all increase with advancing age in human populations^9,10^. This suggests that the ageing process could also exacerbate the effects of programming. This is supported by studies in animal models which demonstrate the importance of age in the phenotypic expression of developmental programming of insulin resistance^11^. Using our rat model of maternal protein restriction during pregnancy followed by normal postnatal nutrition (‘recuperated’), we have previously demonstrated markers of early insulin resistance (including dysregulation of key insulin signalling proteins) in adipose tissue^12,13^ of young first generation (F1) recuperated male offspring. This occurs prior to the development of whole body insulin resistance (as evidenced by hyperinsulinaemia) in first-generation elderly female^14^ and male recuperated offspring^15^. These molecular findings are strikingly similar to the molecular changes observed in adipose tissue^16^ and skeletal muscle^17^ from young adult men who had a low birth-weight suggesting that programming of insulin signalling protein expression is conserved between humans and rodents. Other animal models of developmental programming, including models of food restriction during pregnancy also demonstrate low-birth weight offspring with perturbations in glucose homeostasis and insulin resistance^18^. Interestingly, programmed changes in the offspring can occur in the absence of change in birth weight. For example, individuals born to mothers exposed to the Dutch Hunger Winter during the 1st trimester demonstrated no change in birth weight^19^, despite these offspring having higher adiposity in later life^20^. Furthermore, animal models of glucocorticoid over-exposure during fetal life also demonstrate long term effects without any changes in birth weight^21–23^.

Developmental programming of adverse metabolic phenotypes may also be transmitted to second or subsequent generations of offspring (reviewed in refs^24,25^). The term ‘transgenerational’ in this study has been used, as previously^24^, to denote any effect observed in offspring who were not themselves exposed directly to an adverse maternal diet during pregnancy (also referred to as multigenerational effects)^26^. As the offspring studied here are the F2 generation following an initial dietary modification in the F0 generation, it is possible that any modulations in the physiology of the F2 generation are mediated via the exposed germ-line cells, or via effects on the F1 generation during pregnancy. Epidemiological evidence suggests that children whose grandmothers fasted during Ramadan whilst pregnant tend to have lower birth weights than those whose grandmothers were pregnant during other times of the year^27^. Similarly decreased birth weight, ponderal index and increased incidence of poorer health have all been reported in the F2 of grandmothers exposed to the Dutch Hunger Winter famine whilst pregnant^28,29^. Animal studies have also suggested transgenerational transmission of adverse metabolic phenotypes, including reproductive dysfunction^30,31^, reduced β-cell mass^32,33^ and dysregulation of glucose-insulin homeostasis^34,35^ in the F2 generation. Specifically, Zambrano *et al*. have previously shown transgenerational transmission of whole body insulin resistance at 110 days of age in granddaughters of rat dams exposed to a low-protein diet during pregnancy^36^ and Jimenez-Chillaron *et al*. demonstrated that a mouse model of maternal under-nutrition during pregnancy programs impaired glucose tolerance and impaired β-cell function in the F1 and F2 generations, which may be driven by increased adiposity in these offspring^37^. However, it is not fully understood whether the pathway of molecular changes leading to overt insulin resistance follows a similar course in the second generation as in the first generation in a rat model of protein-restriction.

The aim of this study was therefore to investigate the effect of exposure of grand-maternal protein restriction during pregnancy and accelerated postnatal growth of F1 dams on ovarian adipose tissue morphometry, alterations of insulin signaling and inflammation in adipose tissue and skeletal muscle from the granddaughters (recuperated F2 generation) during both early (3 months) and mid (6 months) adult life.

## Results

### Anthropomorphic measurements

**Figure 1:**
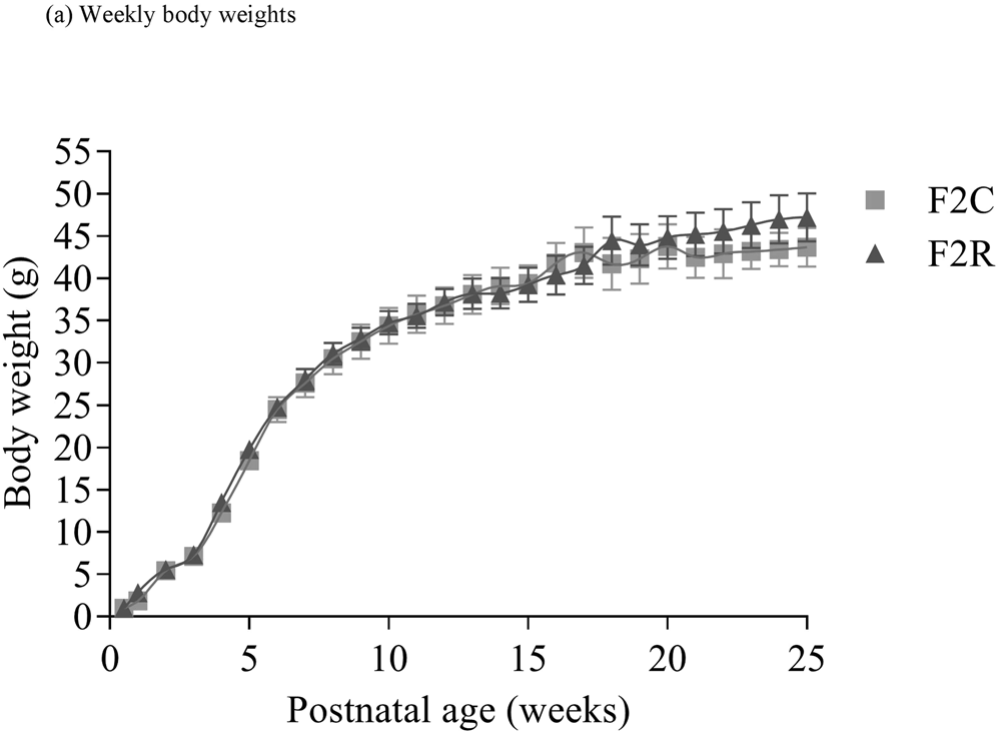
The effect of *in-utero* protein restriction, accelerated postnatal growth and ageing upon postnatal bodyweights of female rats from postnatal week 0 to postnatal week 24. Results are expressed as mean ± S.E.M. C = control; R = recuperated. N values = 7 per group.

There was no significant effect of grand-maternal diet upon body weight (measured weekly from postnatal week 0 to postnatal week 24) (Fig. 1). There was a significant (p < 0.05) overall effect of grand-maternal diet upon para-ovarian fat pad weight, with the increase in para-ovarian fat pad weight in the F2 recuperated offspring being much greater at 6 months of age (control; 4.4 ± 1.1 g, recuperated; 6.9 ± 2.6 g) than at 3 months of age (control 2.8 ± 0.7 g; recuperated 2.9 ± 0.8 g). There was no significant effect of age or grand-maternal diet upon fasting plasma glucose (FPG) or fasting insulin levels at this age (Table 1). There was no significant effect of grand-maternal diet upon mean adipocyte cell size (control 3 m; 4673 ± 420 µm^2^, recuperated 3 m; 4469 ± 682 µm^2^, control 6 m; 5494 ± 890 µm^2^, recuperated 6 m; 5763 ± 577 µm^2^), however there was a trend (p = 0.09) to suggest that adipocyte cell size increased with age. More detailed analysis dividing adipocytes into small and large adipocytes revealed for control F2 animals there was a similar percentage of small adipocytes between 3 and 6 months of age (19.7 ± 2.2% vs. 20.3 ± 2.2%). However the recuperated F2 animals showed a significant reduction in the number of small adipocytes between 3 and 6 months of age (22.6 ± 2.3% vs. 11.8 ± 1.8%, p < 0.01) (Supplementary Figure 1, S1).

**Table 1:** Fasting plasma glucose and fasting serum insulin measurements.

Group	Fasting plasma glucose (mmol/L)	Fasting serum insulin (ng/ml)
F2C 3 m	5.5 ± 0.1	1.1 ± 0.1
F2R 3 m	5.5 ± 0.1	0.8 ± 0.09
F2C 6 m	5.6 ± 0.5	1.0 ± 0.1
F2R 6 m	6.5 ± 0.4	1.0 ± 0.1

F2C = F2 Control.

F2R = F2 Recuperated.

The effect of in-utero protein restriction, accelerated postnatal growth and ageing upon fasting plasma glucose and fasting serum insulin measurements in 3 and 6 month female rats. Results are expressed as mean ± S.E.M. F2C = control F2; F2R = recuperated F2; N = 7 per group.

### Para-ovarian fat insulin signalling cascade dysregulation is transmitted to the F2 generation

**Figure 2:**
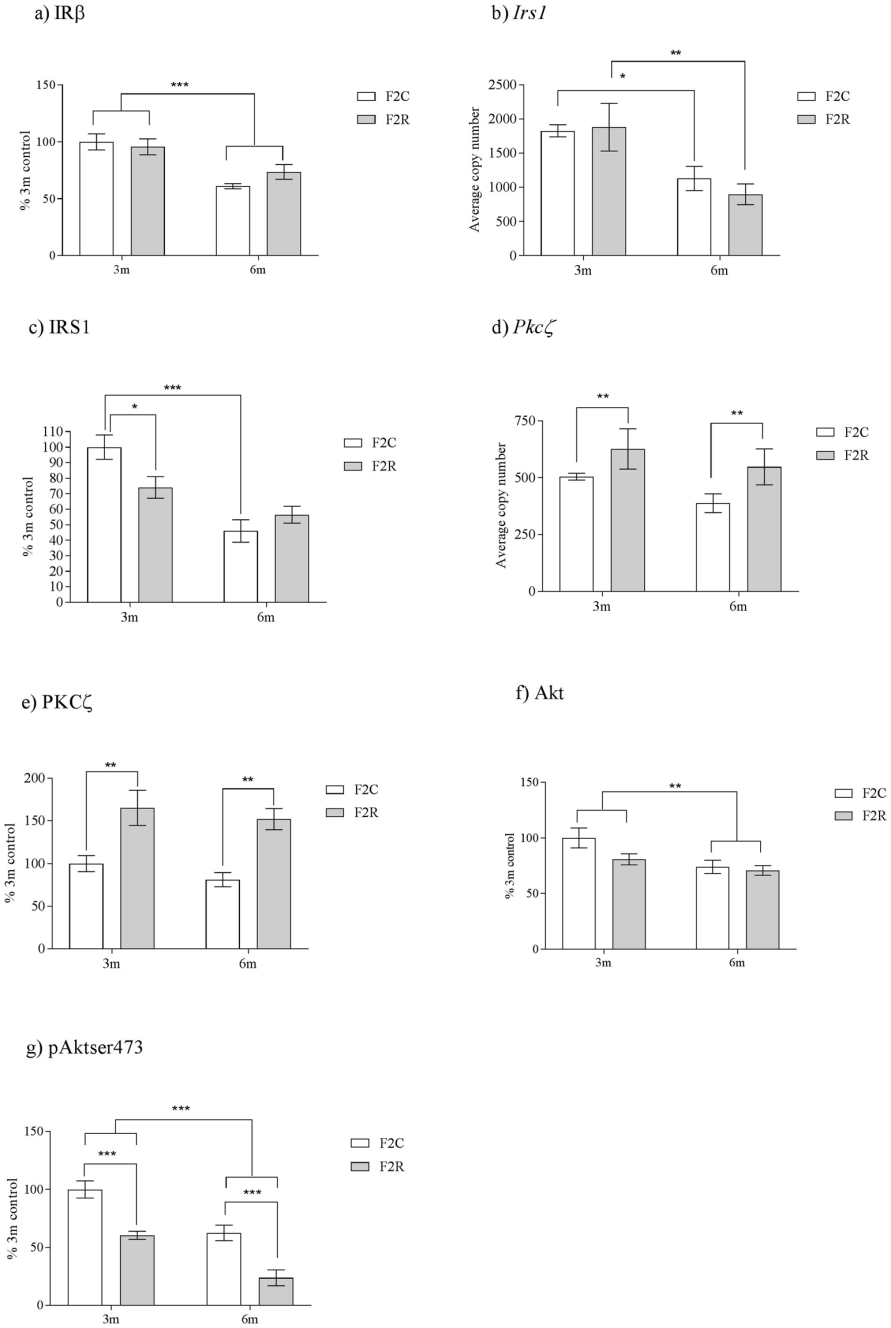
The effect of *in-utero* protein restriction, accelerated postnatal growth and ageing upon expression of insulin signaling molecules in para-ovarian fat of 3 and 6 month female rats (**a**) IRβ protein expression (**b**) pIRβ^Y1361^ protein expression, (**c**) *Irs1* mRNA expression (**d**) IRS1 protein expression (**e**) *Pkc-ζ* mRNA expression, (**f**) PKC-ζ protein expression, (**g**) Total Akt protein expression and (**h**) pAkt^ser473^ protein expression. Results are expressed as mean ± S.E.M. F2C = control; R = recuperated. *p < 0.05, **p < 0.01 and ***p < 0.001. N values: mRNA analysis = 7 per group. Protein analysis: 3 m C = 7, 3 m recuperated = 7, 6 m control = 6, 6 m recuperated = 5.

IRβ protein expression was unaltered by grand-maternal diet, however there was a significant (p < 0.001) overall effect of age upon IRβ, with IRβ decreasing with age (Fig. 2a). The mRNA expression of Insulin receptor substrate-1 (*Irs-1*) was unchanged between groups (Fig. 2b). However, at 3 months of age, adipose tissue IRS-1 protein expression was decreased (p < 0.05) in F2 R, compared to F2 C (Fig. 2c). These data indicate a post-transcriptional modification of IRS-1 expression at 3 months of age. By 6 months of age, mRNA and protein expression of IRS-1 were unchanged between groups (Fig. 2b,c). There was a significant (p < 0.001) overall effect of age upon both IRS-1 mRNA and protein (p < 0.01) with IRS1 expression decreasing with age. Phosphatidylinositol-3-kinase (P110-β) mRNA and protein expression were not affected by grand-maternal diet or age (3 m control; 100 ± 4, 3 m recuperated; 115 ± 10, 6 m control; 101 ± 7; 6 m recuperated 92 ± 9) (% 3 m control). There was an overall effect of grand-maternal diet upon *Pkc*-ζ mRNA expression (p < 0.05) and protein kinase C zeta (PKC-ζ) protein (p < 0.001), with PKC- ζ expression increased (p < 0.01) in F2 R at both 3 and 6 months of age (Fig. 2d,e) compared to F2 C. There was no effect of age upon PKC- ζ mRNA or protein expression (Fig. 2d,e). There was no overall effect of grand-maternal diet upon total Akt protein expression (Fig. 2f). However, total Akt protein expression was significantly (p < 0.01) reduced between 3 and 6 months of age (Fig. 2f). There was also a robust effect of grand-maternal diet upon protein expression of phosphorylation of Akt which demonstrated significantly (p < 0.001) decreased pAkt^ser473^ in recuperated F2 animals at both 3 months and 6 months of age (Fig. 2g). pAkt^ser473^ was also significantly (p < 0.001) reduced between 3 and 6 months of age in control and recuperated F2 animals (Fig. 2g).

### Vastus lateralis (VL) skeletal muscle insulin signaling cascade regulation occurs in the F2 generation

**Figure 3:**
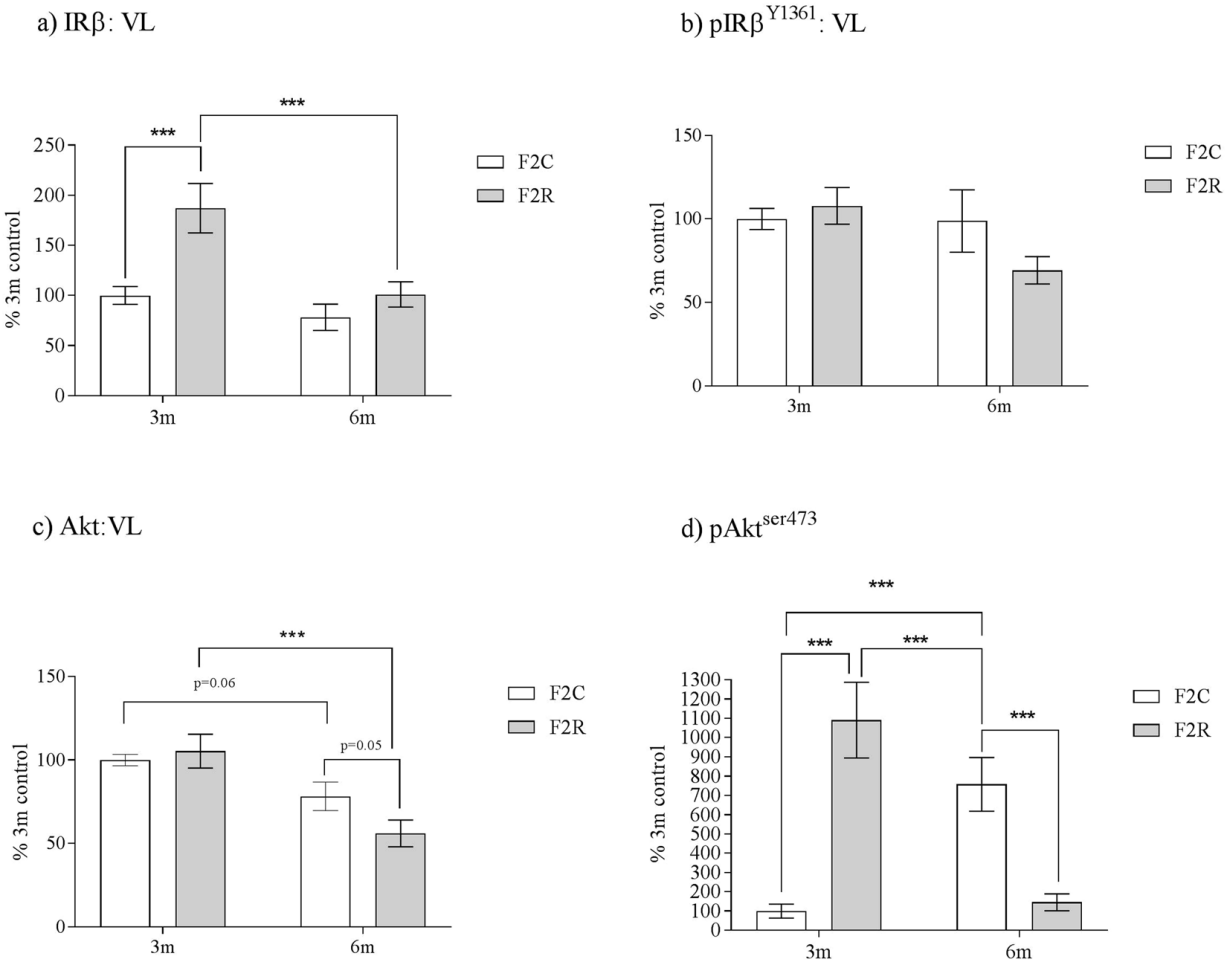
The effect of *in-utero* protein restriction, accelerated postnatal growth and ageing upon VL skeletal muscle protein expression of (**a**) IRβ, (**b**) pIRβ^Y1361^ (**c**) Akt and (**d**) PAkt^ser473^ in 3 and 6 month female rats. Results are expressed as mean ± S.E.M. **p < 0.01. C = control; R = recuperated. N values: mRNA analysis = 7 per group. Protein analysis = 6 per group.

There was a significant interaction between grand-maternal diet and age on insulin receptor (IRβ) expression (p < 0.05). This reflected an increased (p < 0.001) protein expression of IR-β in F2 R VL muscle compared to F2 C at 3 months but not at 6 months (Fig. 3a). Additionally, protein expression of the phosphorylated form of IR-β (IR-β^Y1361^) was unaffected by maternal diet and age (Fig. 3b). There was no effect of maternal diet upon protein expression of total Akt in VL skeletal muscle, however Akt protein expression decreased with age (p < 0.001) (Fig. 3c). This was accompanied by a highly significant (p < 0.001) interaction between grand-maternal diet and age upon phosphorylation of Akt (pAkt^ser473^). This demonstrated that phosphorylation of pAkt^ser47^ was significantly (p < 0.001) increased in F2 R at 3 months of age, however, at 6 months of age, F2 R had significantly (p < 0.001) less PAkt^ser473^ compared to F2 C (Fig. 3d).

### Alterations of pro-inflammatory marker levels in adipose tissue are transmitted to the F2 generation

**Figure 4:**
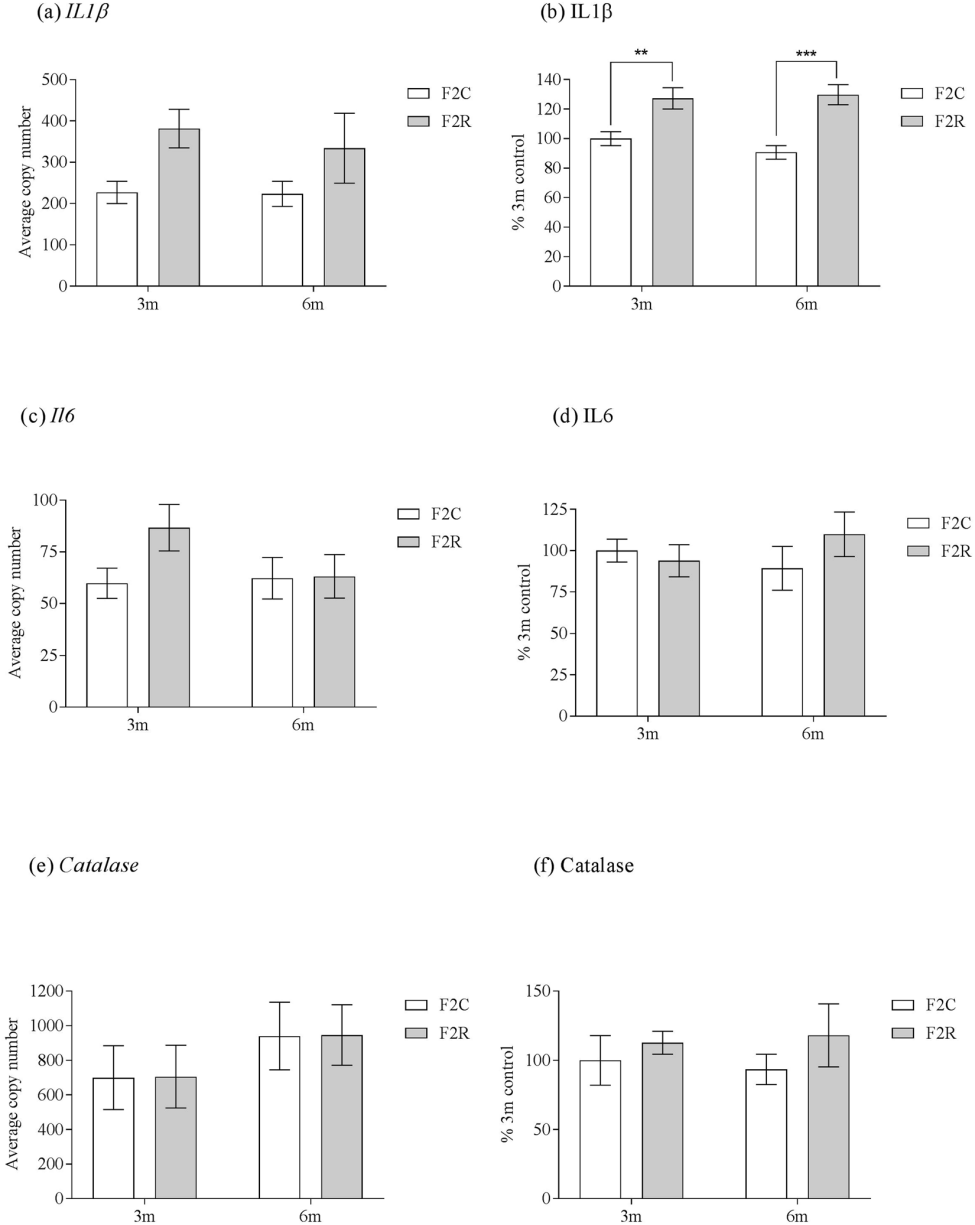
The effect of *in utero* protein restriction, accelerated postnatal growth and ageing upon expression of pro-inflammatory molecules in para-ovarian fat of 3 and 6 month female rats (**a**) *Il1-β* mRNA expression (**b**) IL1-β protein expression, (**c**) *Il-6* mRNA expression (**d**) IL-6 protein expression, (**e**) *Catalase* mRNA expression and (**f**) Catalase protein expression. Results are expressed as mean ± S.E.M. **p < 0.01, ***p < 0.001. N values: mRNA analysis = 7 per group. Protein analysis: 3 m control = 7, 3 m recuperated = 7, 6 m control = 6, 6 m recuperated = 5.

There was an overall effect of grand-maternal diet (p < 0.05) upon *Il1-β* mRNA expression, with *Il1-β* mRNA expression being increased in F2 R compared to F2 C (Fig. 4a). There was no significant effect of age upon IL-1β protein or mRNA expression (Fig. 4a,b). There was also a significant (p < 0.001) effect of grand-maternal diet upon interleukin-1β (IL-1β) protein expression, with increased IL-1β protein levels in the F2 R at 3 months (p < 0.01) and 6 months (p < 0.001) of age (Fig. 4b). There was no significant effect of grand-maternal diet or age upon *Il-6* and *Catalase* mRNA or interleukin-6 (IL-6) and Catalase protein expression (Fig. 4c,e and f).

## Discussion

Epidemiological and animal studies have established that low-birth weight and accelerated postnatal catch-up growth increases risk of development of age-associated metabolic disease in later life including insulin resistance and type-2 diabetes, (reviewed in^38^). Previously, using our maternal protein-restriction model, we reported that F1 recuperated male rat offspring had low-birth weight, underwent accelerated postnatal catch-up growth and had unaltered fasting plasma glucose levels in young adult life^12,13^ which was also recapitulated in female F1 recuperated offspring^39^. Male F1 recuperated offspring also demonstrated dysregulated adipose tissue insulin signalling,^12,13^, alterations in adipocyte size^12^, and an increase in inflammatory cytokines in adipose tissue^13^ at 3 months of age. These tissue level changes are the direct antecedents of developing insulin resistance, and occur before whole-body insulin resistance is discernible.

Adipose tissue is a major contributor to whole body insulin sensitivity^40^ and this tissue is particularly vulnerable to the effects of developmental programming^12,41,42^. Although para-ovarian fat pad mass was increased in F2 recuperated animals, no effect of grand-maternal diet upon adipocyte cell size was observed. However, the adipocyte cell size distribution data demonstrated a significant reduction in the number of small adipocytes between 3 and 6 months of age in F2 recuperated animals, which was not observed in the control F2 group. This cell size distribution shift with age may go some way to explain the increased para-ovarian pad mass finding in F2 recuperated animals. On a molecular level, we observed reduced IRS-1 protein expression in para-ovarian adipose tissue in the F2 recuperated group at 3 months of age. *Irs1* mRNA expression was similar between grand-maternal dietary groups at 3 months of age, suggesting post-transcriptional dysregulation of IRS-1 levels which is consistent previous observations in the F1 recuperated group at 3 months^12,13^. The mechanisms underlying this post-transcriptional change are unknown. However we have shown previously in F1 recuperated adipose tissue it is not related to miRNAs known to regulate IRS-1^13^. Previous studies have established that loss of IRS-1 is associated with insulin resistance and T2D: low levels of IRS-1 have been observed in adipocytes of individuals with frank diabetes^43^ and IRS-1 null mice are growth restricted with evidence of peripheral insulin resistance^44^. Our observation of an early reduction in IRS-1 expression in the second generation of recuperated animals in this study thus may be the first step towards the development of insulin resistance in this group. PKC-ζ protein and mRNA expression were both increased in the F2 recuperated group, consistent with our previous findings in the F1 generation^12,13^. Up-regulation of PKC-ζ can negatively regulate insulin signalling via serine phosphorylation of IRS-1^45^. Hence the synergistic effect of both reduced expression and increased inactivation of IRS-1 could be a precursor to insulin sensitivity in the F2 recuperated animals, however given the difficulty of finding a suitable pIRS-1^ser312^ antibody, this remains at present, speculative. We previously demonstrated decreased protein expression of P110-β, the catalytic subunit of phosphoinositide 3′-kinase (PI3-kinase) in the adipose tissue of the F1 generation, however in the F2 generation there was no difference in P110-β levels between groups, suggesting that modulation of P110-β expression is not sensitive to transgenerational transmission. Total Akt protein expression was unaltered by grand-maternal diet, however we observed a robust decrease in the phosphorylation of Akt at the ser^473^ residue, which is consistent with what we observed in the F1 generation at 3 months of age^12^. This, once again may suggest some measure of insulin resistance in the adipose tissue. Indeed, it has been shown that basal levels of pAkt_ser473_ were decreased in the adipose tissue of obese patients with insulin resistance^46^.

Increased inflammation is known to be an important factor in the aetiology of insulin resistance and type-2 diabetes^47,48^. Previously, we reported increased protein levels of the pro-inflammatory cytokines IL1β and IL6 in F1 recuperated offspring adipose tissue compared to F1 controls^12^. In this study, we found increased IL1β protein levels in the adipose tissue of F2 recuperated offspring compared to F2 controls, demonstrating that a pro-inflammatory phenotype is maintained in the F2 generation. IL6 protein and mRNA levels were unchanged in the adipose tissue F2 recuperated offspring, suggesting that only IL1β is sensitive to transgenerational transmission of phenotype.

The largest proportion (60–70%) of insulin-mediated glucose uptake occurs within the skeletal muscle and this is mediated by insulin action on insulin receptors in the plasma membrane. At 3 months of age, protein expression of IR-B was significantly increased (2-fold) in the VL skeletal muscle of F2 recuperated offspring compared to controls, a potential marker of increased insulin sensitivity. This observation mirrors closely our previous findings of F1 low-protein male offspring^17,49,50^. IR-β protein expression in VL skeletal muscle was not different at 6 months between F2 recuperated and F2 control offspring, consistent with a decline in insulin sensitivity with age in recuperated offspring. This may suggest that, despite initial increased insulin sensitivity at 3 months of age, this has disappeared by 6 months of age. Consistent with increased skeletal muscle IR protein expression (and the potential for insulin sensitivity) at 3 months of age in recuperated F2, phosphorylated Akt(^ser473^) was also increased in VL skeletal muscle. However, by 6 months of age, a 7-fold age-associated decrease in pAktser^473^ occurred in the F2 recuperated offspring, which resulted in levels being significantly reduced compared to controls. It has been shown in *db/db* mice, (a model of insulin resistance, diabetes and obesity), that maximal Akt^ser473^ phosphorylation was decreased by 32% in skeletal muscle, which corresponded with a significant decrease in maximal Akt kinase activity^51^ and muscle cells from diabetic patients were insulin defective and this was associated with reduced PAkt^ser473^ ^52^. The observed reduction of pAkt^ser473^ was accompanied by no change in expression of total Akt protein which is also consistent with the findings in *db/db* mice^52^. However, it must be noted that as our measurements were performed in the basal state, in the absence of insulin, therefore it is possible that under insulin stimulation, Akt phosphorylation may increase in both groups, independent of the basal state.

The mechanism by which the metabolic phenotype of the F2 generation is influenced by a suboptimal diet during grand-maternal pregnancy remains a matter of interest. The major mechanism proposed include (i) a direct impact of grand-maternal diet on the F2 generation, for example by epigenetic modification of the oocyte or reprogramming effects on the ooplasm and (ii) an indirect impact via a suboptimal reproductive tract environment or poor maternal adaptation to pregnancy in the F1 generation. We have previously reviewed the evidence for these mechanisms^21,22^. While evidence strongly suggests that trans-generational developmental programming via the paternal line may be mediated by epigenetic modification of the spermatozoa^21^, transmission via the maternal line has many more potential mechanisms. A limitation of our current study is that the data do not enable us to further disambiguate the precise mechanism driving the observed effects.

In conclusion, our findings suggest that grand-maternal protein restriction causes alterations in insulin signalling molecules in skeletal muscle and adipose tissue in the F2 generation. Taken together, we suggest that F2 adipose tissue is more vulnerable to the effects of developmental programming at a young age and that this tissue is an early site of origin of the development of insulin resistance in this model, as was observed in F1 tissues^12,13^. This is highlighted by our observations at 3 months of age in F2 adipose tissue displaying reduced IRS-1 and reduced pAkt^ser473^, which were both consistent with insulin resistance in this tissue. In contrast, 3 month VL skeletal muscle tissue had increased IRβ and increased pAkt^ser473^, consistent with a (compensatory) increase in insulin sensitivity. At 6 months of age both tissues demonstrated decreased pAkt^ser473^, suggestive of insulin resistance in both tissues. In both cases, these molecular changes may contribute to an increased risk of whole body insulin resistance in later life.

## Methods

All protocols were approved by the animal welfare ethical review process of the University of Cambridge and carried out under licence from the U.K. Animals (Scientific Procedures) Act 1986. Stock animals were purchased from Charles River. Dams were produced from in-house breeding of stock animals. Pregnant Wistar rats (*rattus norvegicus*) were maintained at 22 °C, on a controlled 12:12-h light-dark cycle, in specific pathogen free (SPF) housing using individually ventilated cages with environmental enrichment. The dams were maintained on a 20% protein diet (control) or, an isocaloric low protein (LP) (8%) diet, as previously described^53^. The percentage composition of the diets (dry weight) can be seen in Supplementary Table 1, (T1). Access to diets and water was provided *ad libitum*. Diets were purchased from Arie Blok (Woerden, The Netherlands).

The day of birth was recorded as day 1 of postnatal life. Pups born to LP diet-fed dams were cross-fostered to control-fed mothers on postnatal day 3, in order to create a recuperated litter. Each recuperated litter was standardized to 4 female pups at random to maximize their plane of nutrition. The control group was the offspring of mothers fed the 20% protein diet and suckled by 20% protein fed dams. Each control litter was culled to 8 female pups as a standard. This group were suckled (in litters of 8) by their own dams. To minimize stress to the animals when cross-fostered, pups were transferred with some of their own bedding. After weaning (at 22 days of age), all first-generation offspring were maintained on standard laboratory chow *ad-libitum*. At 12 weeks of age, the female first-generation (F1) were mated with stock males to produce a second generation of offspring (F2). The second generation were suckled by their own mothers (8 pups per mother) and weaned onto on a standard laboratory chow fed *ad-libitum* at 21 days of age (diet from Arie Blok, Woerden, The Netherlands), and therefore were not directly exposed to any nutritional insult. Body weights were recorded weekly from birth to 24 weeks of age. For time points up until 3 weeks of age these reflect average female pup weight in the litter. One female F2 per litter was culled at 3 months of age, and a second female was culled at 6 months of age. All animals were killed by CO_2_ asphyxiation at approximately 10 am after an overnight fast. At post-mortem, para-ovarian fat pad tissue and vastus lateralis skeletal muscle was removed, weighed, and snap frozen in liquid nitrogen and then stored at −80 °C until analysis. A portion of para-ovarian fat (taken from the same place for each sample) was removed and fixed in 10% neutral buffered formalin (NBF) for histological assessment. Seven litters per group were used in this study. In all cases, n refers to the number of litters (with 1 animal used from each litter at each time point).

### Fasting plasma glucose and fasting serum insulin measurements

Fasted blood glucose measurements were obtained using a blood glucose analyser (Hemocue, Angelholm, Sweden). Serum was obtained from blood collected from the tail vein after overnight fasting. The blood was left to clot for 30 minutes before centrifugation for 3 minutes at 845 × g. Fasting serum insulin measurements were performed using a commercial kit (Crystal Chem, Zaandam, Netherlands).

### Protein expression

Protein was extracted from whole tissue lysates of para-ovarian fat tissue and vastus lateralis skeletal muscle and assayed as described previously^13^. Protein (20μg) was loaded onto 10%, 12% or 15% polyacrylamide gels, dependent upon the molecular weight of the protein to be measured. The samples were electrophoresed and transferred to polyvinylidene fluoride membranes^13^, and detected using the following primary antibodies: IRβ (1:200); Santa Cruz, Wembley, Middlesex, UK)^11^, pIRβ^Y1361^ (1:1000); Abcam, Cambridge, Cambs, UK, IRS-1 (1:1000); Merck-Millipore, Watford, Herts, UK^11^, (PKC-ζ (1:200); Santa-Cruz, Wembley, Middlesex, UK)^12^, pAkt^ser473^ (1:1000); New England Biolabs, Hitchin, Herts, UK, P110-β (1:1000); Santa-Cruz, Wembley, Middlesex, UK^12,13^, IL1-β (1:1000); Abcam, Cambridge, Cambs, UK, IL6 (1:1000); Abcam, Cambridge, Cambs, UK^15^, and Catalase (1:10000); Abcam, Cambridge, Cambs, UK^15^. All primary antibodies required anti-rabbit IgG secondary antibodies, except total Akt. Total Akt required an anti-mouse IgG secondary antibody. All secondary antibodies were purchased from Cell Signaling Technology, Danvers, MA, USA, and were used at a dilution of 1:2000. Equal protein loading was confirmed by staining electrophoresed gels with Coomassie Blue (Bio-Rad, Hemel Hempstead, Herts, UK) to visualize total protein (Supplementary Figure 2, S2). To ensure that the chemiluminescent signal changed in a linear manner, the ratio between loading controls (100% and 50% pooled sample) was confirmed for each detected protein [as detailed as S1 and S1 (50%)]. All Western blot full length images used for analysis are to be found as Supplementary Figure 3, S3.

### Gene expression

RNA was extracted using an RNeasy Plus mini kit (Qiagen, Manchester, Lancs, UK) following manufacturers’ instructions. A DNase digestion step was performed in order to ensure no gDNA contamination. RNA (1 µg) was used to synthesize cDNA using oligo-dT primers and M-MLV reverse transcriptase (Promega, Southampton, Hants, UK). Gene expression was determined using custom designed primers (Sigma, Poole, Dorset, UK) and SYBR Green reagents (Applied Biosystems, Warrington, Cheshire, UK). Primer sequences are presented in Table 2. Quantification of gene expression was performed using a Step One Plus RT-PCR machine (Applied Biosystems, Warrington, Cheshire, UK). Equal efficiency of the reverse transcription of RNA from all groups was confirmed through quantification of expression of the housekeeping gene *ppia*. Expression of *ppia* did not differ between groups.

**Table 2:** Primer table.

Primer	Sequence (F) 5′- 3′	Sequence (R) 5′-3′
*Irs1*	TGGCAGTGAGGATGTGAAAC	CTTGGATGCTCCCCCTAGAT
*P110β*	TGAGGTTGTGAGCACCTCTG	CTTTGTTGAAGGCTGCTGTG
*Pkcζ*	GGGTGGATGGGATCAAAATC	GGAGGACCTTGGCATAGCTT
*Il1β*	TGGAAAAGCGGTTTGTCTTC	TGCTTGAGAGGTGCTGATGT
*Il6*	TACCCCAACTTCCAATGCTC	GTTGGATGGTCTTGGTCCTT
*Catalase*	TTGGATCATGTCTTCCAAAAA	GGGAAAAGGAATCCGATCAA
*Ppia*	GCAAGTCCATCTACGGAGAGA	TGTGTTTGGTCCAGCATTTG

Primer sequence table for primers utilised in this study.

### Para-ovarian fat pad measurement

A biopsy of para-ovarian tissue was obtained and fixed in 10% formalin/paraldehyde. Fixed adipose tissue was embedded and stained with haematoxylin and eosin (H&E). Three section levels were obtained from each animal. The slides were scanned and the images analysed using Zen Lite software (Carl Zeiss AG, Germany). A single blinded observer assessed all slides to prevent bias in the analysis. 12 separate fields of view from each whole-image slide were used to assess adipocyte size, which was measured to the nearest µm^2^. The fields for analysis were selected using a randomly placed grid. The results were analysed using a hierarchical logistic regression model, with fixed effects for offspring age and grand-maternal diet. Random effects in the model were included for each animal and each litter-of-origin.

### Equipment and settings

All western blotting images were scanned using a Sharp MX-3570 scanner (Sharp, London, UK) set at 300 dpi, using the colour setting. All images were saved as JPEGs for analysis. All images were analysed using Alpha Ease densitometry software (Alpha Ease, Alpha Innotech, San Leandro, CA, USA).

### Statistical analysis

Data were analysed either using a 2-way ANOVA with grand-maternal diet and offspring age as the independent variables and Duncan’s Post-Hoc testing where appropriate or by a repeated measures ANOVA, in the case of the pre-weaning body weight data. Data are represented as mean ± S.E.M. A value of p < 0.05 was considered statistically significant. All data passed normality testing. All statistical analyses were performed using Statistica 7 software (Statsoft Inc, Milton Keynes, Buckinghamshire, UK). In all cases, N refers to the number of litters.

### Data availability statement

Authors confirm that all materials and methods will be available to others to replicate these data.

## Electronic supplementary material

Supplementary data
