# Supplementary material for: Insulin-signalling dysregulation and inflammation is programmed trans-generationally in a female rat model of poor maternal nutrition
Source: Sci Rep. 2018 Mar 5;8:4014. doi: 10.1038/s41598-018-22383-w (PMC5838091; doi:10.1038/s41598-018-22383-w)
Supplement: Supplementary file 1 — Supplementary data [file 41598_2018_22383_MOESM1_ESM.pdf]

Supplementary data

Insulin-signalling dysregulation and inflammation is programmed trans-generationally in a female rat model of poor maternal nutrition.

Authors: Jane L. Tarry-Adkins[1]*, Catherine E. Aiken[1], Thomas J. Ashmore[1] and Susan E. Ozanne[1].

University of Cambridge Metabolic Research Laboratories and MRC Metabolic Diseases Unit, Wellcome Trust-MRC Institute of Metabolic Science, Level 4, Box 289, Addenbrookes' Treatment Centre, Addenbrookes' Hospital, Hills Road, Cambridge, CB2 OQQ, UK.

[1]University of Cambridge Metabolic Research Laboratories and MRC Metabolic Diseases Unit, Wellcome Trust-MRC Institute of Metabolic Science, Level 4, Box 289, Addenbrookes' Treatment Centre, Addenbrookes' Hospital, Hills Road, Cambridge, CB2 OQQ, UK

Phone: +(44) 1223 336784, Fax: +(44) 1223 330598. Email : jlt28@cam.ac.uk

Supplementary Figure S1: Adipocyte cell size distribution data

A) Control 3m vs. 6m

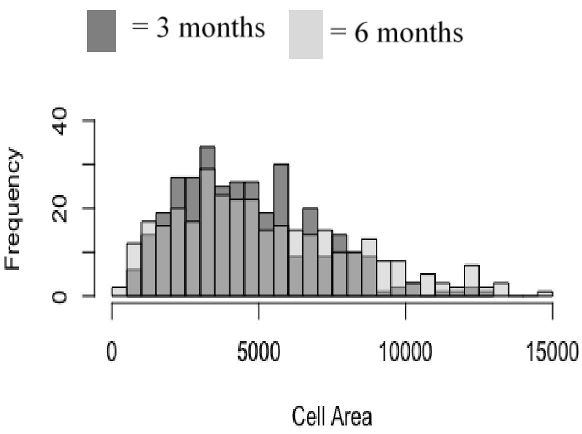

B) Recuperated 3m vs. 6m

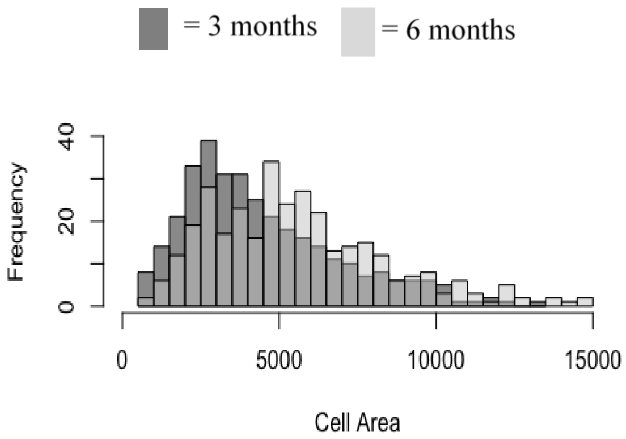

Supplementary Figure S2: Commassie staining for equal loading of protein samples

a) Ovarian fat

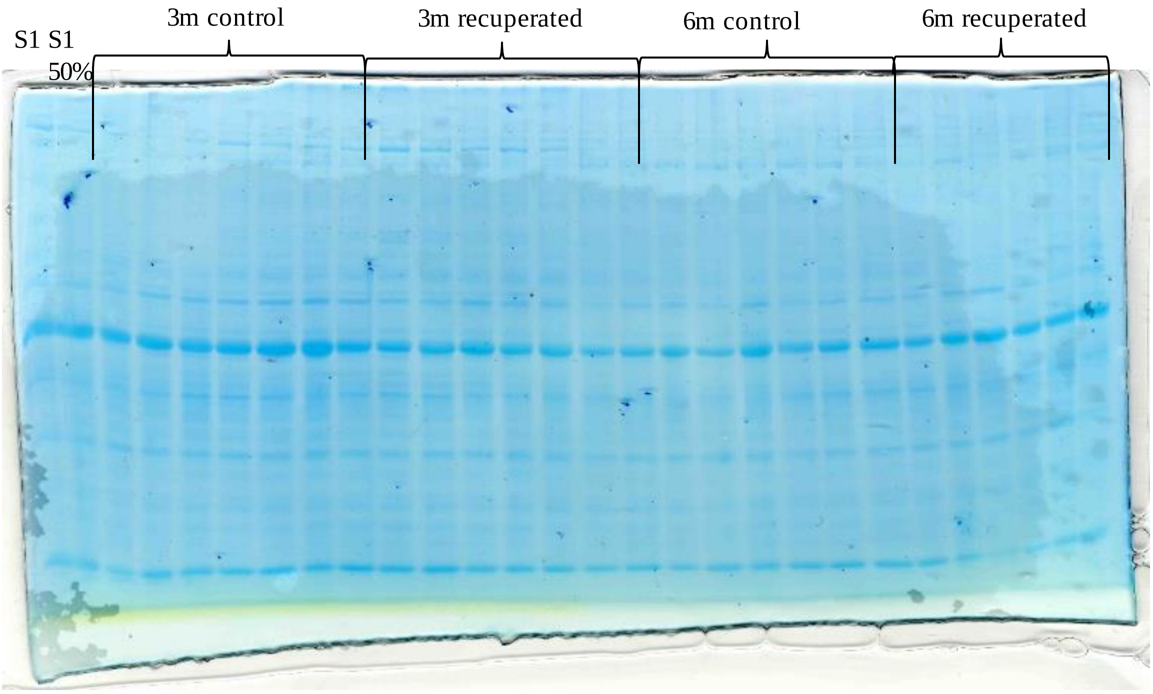

b) VL skeletal muscle

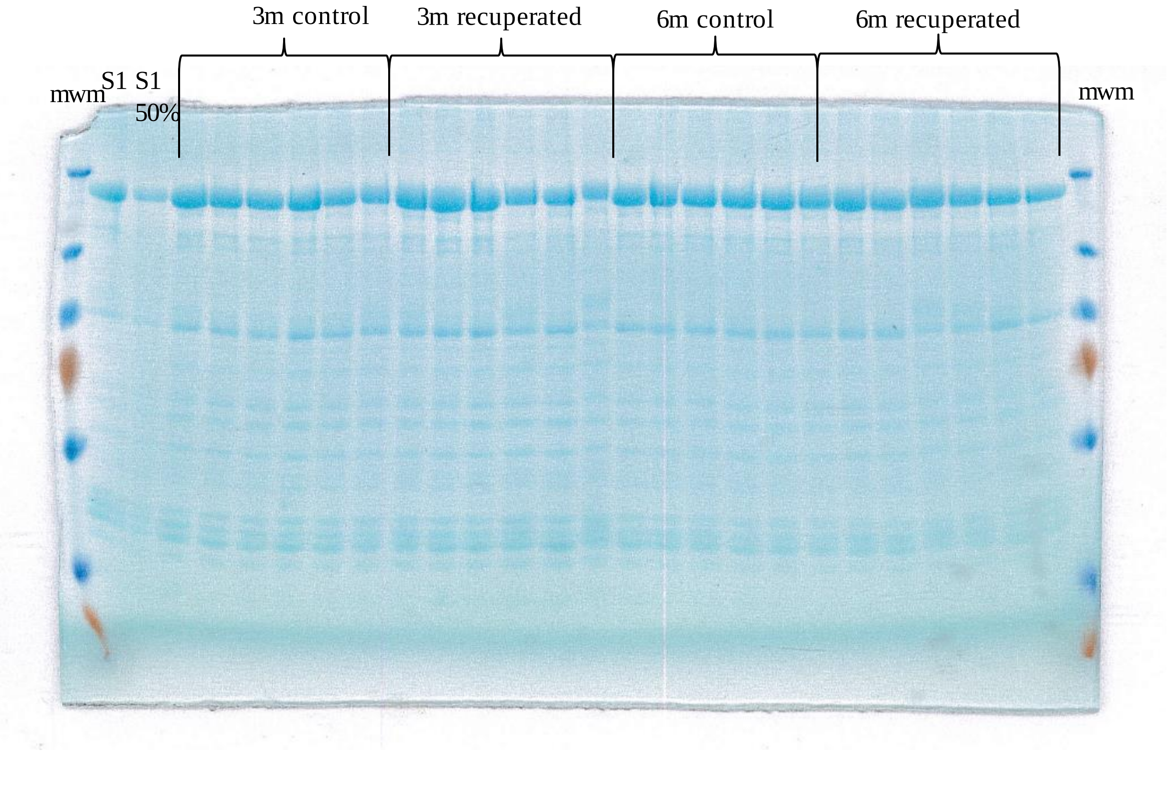

Supplementary Figure 3 S3: Full-length gel images for Western blot analysis

Figure 2

a) 3 & 6m Ov fat (IRβ)

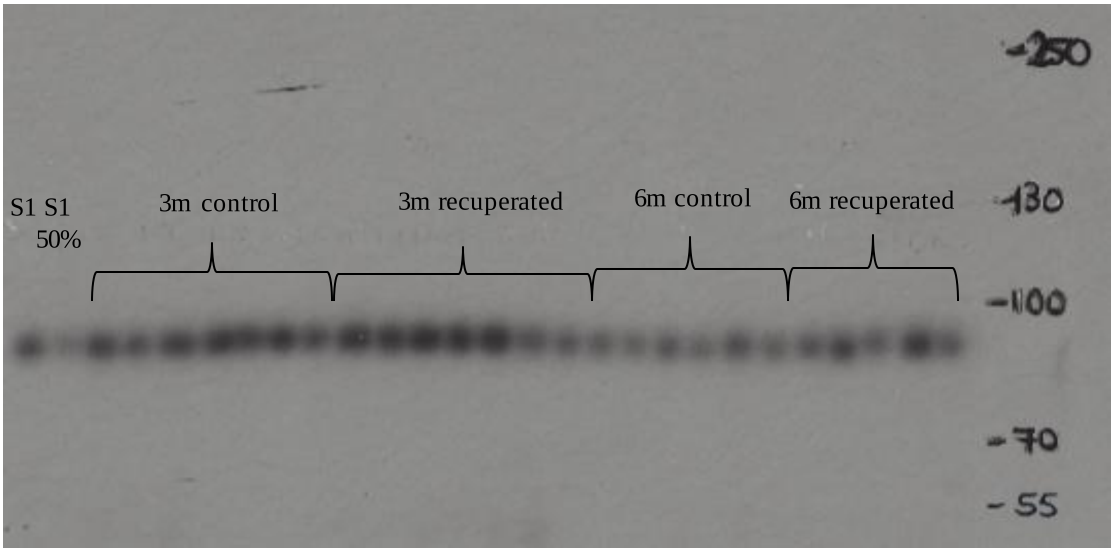

c) 3 & 6m Ov fat (IRS1)

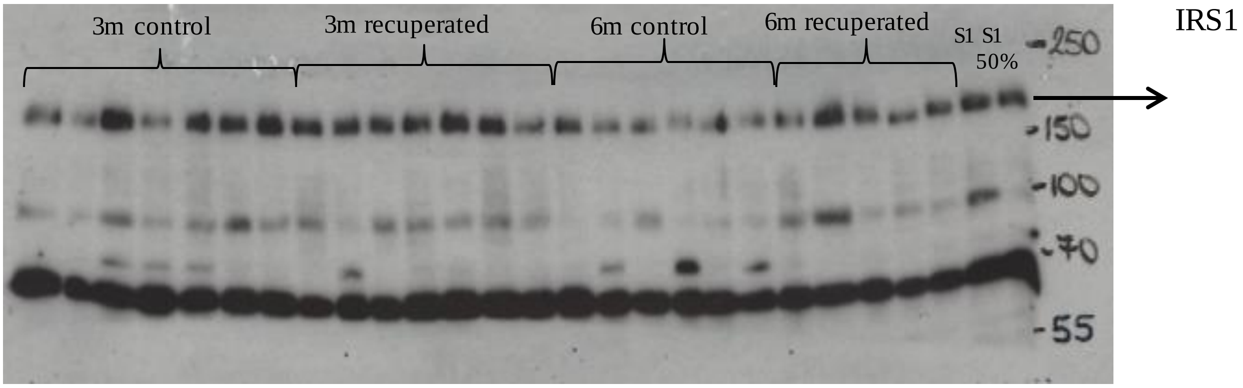

e) 3 & 6m Ov fat (PKCz)

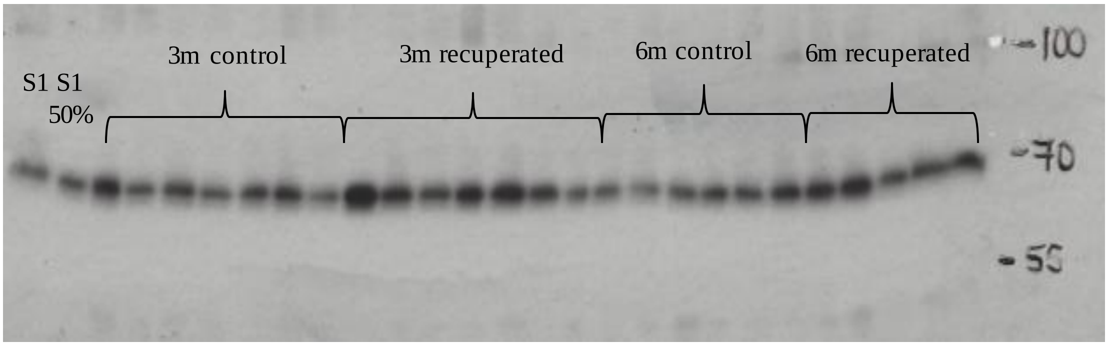

Figure 2 (continued)

f) Ov fat (Akt)

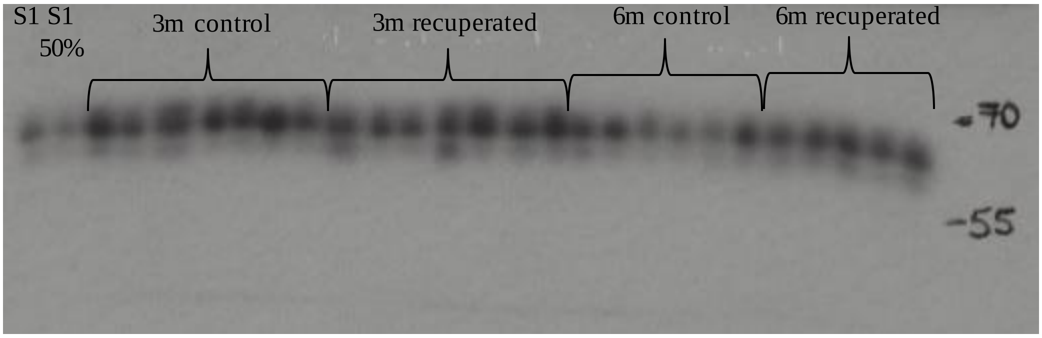

g) Ov fat (PAkt^{Ser374})

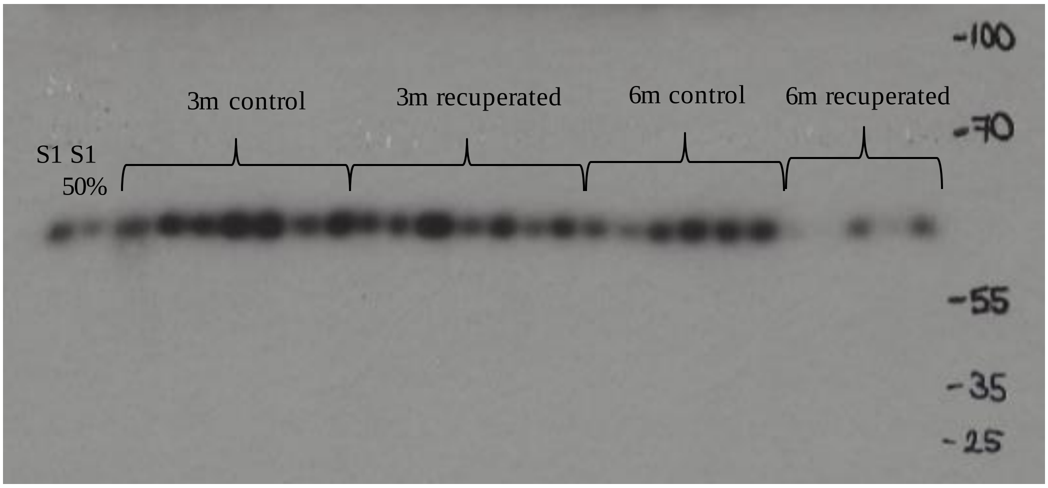

h) Ov fat (P110β) (1 min exposure time)

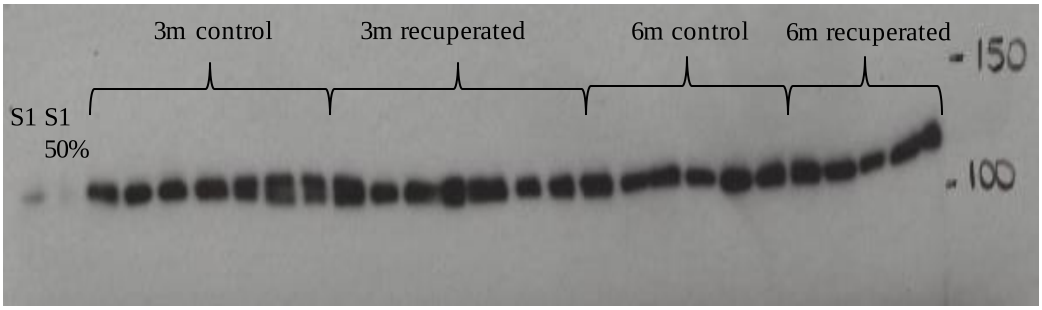

i) Ov fat (P110β) (30 sec exposure time)

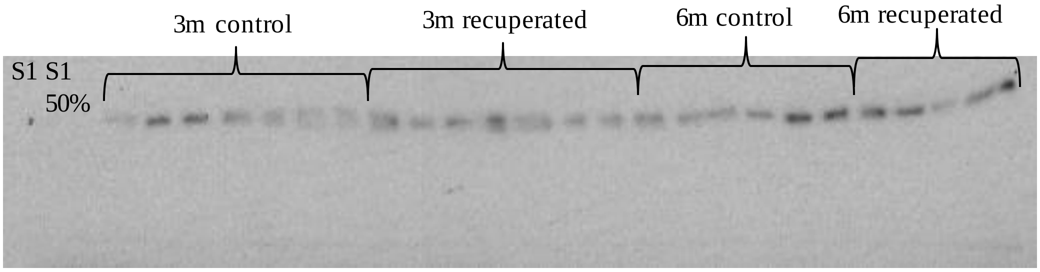

Figure 3

a) IRβ: VL

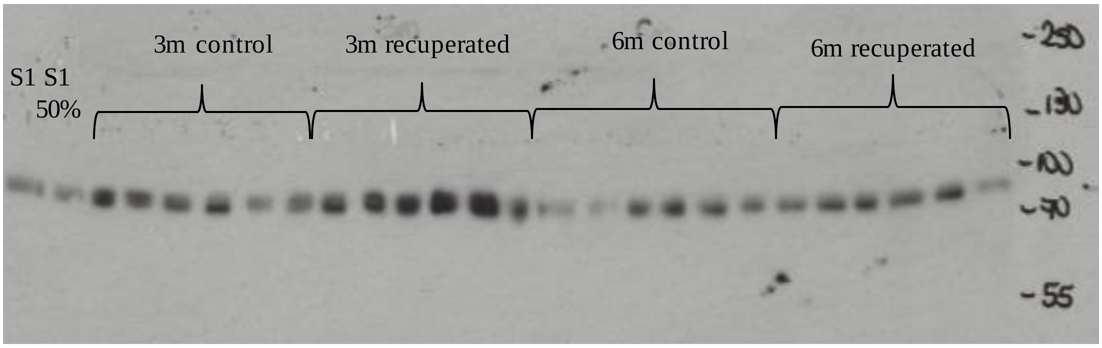

b) PIRβ^{Y1361}: VL

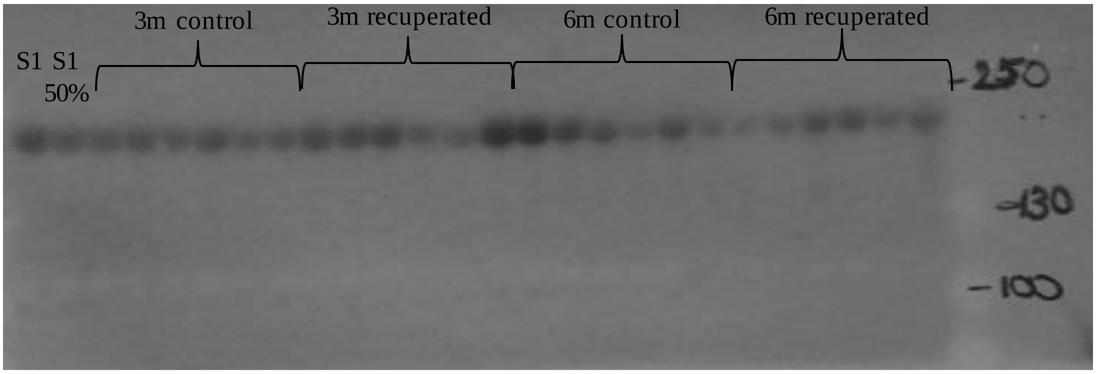

c) Akt: VL

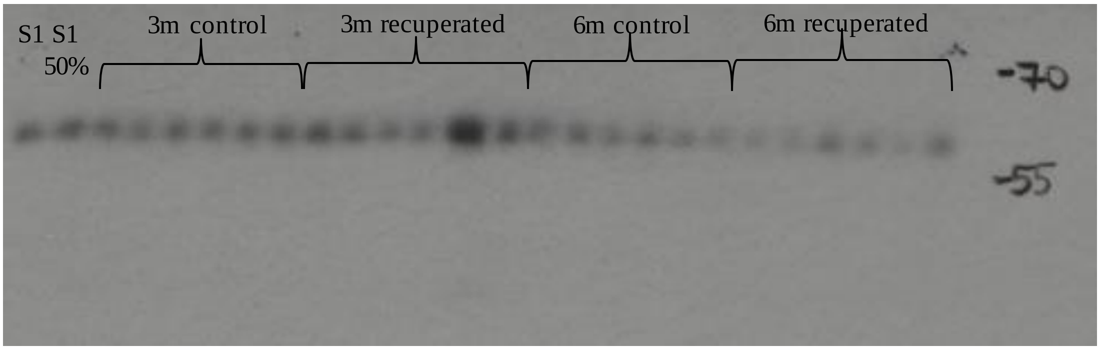

d) pAkt^{ser473}: VL

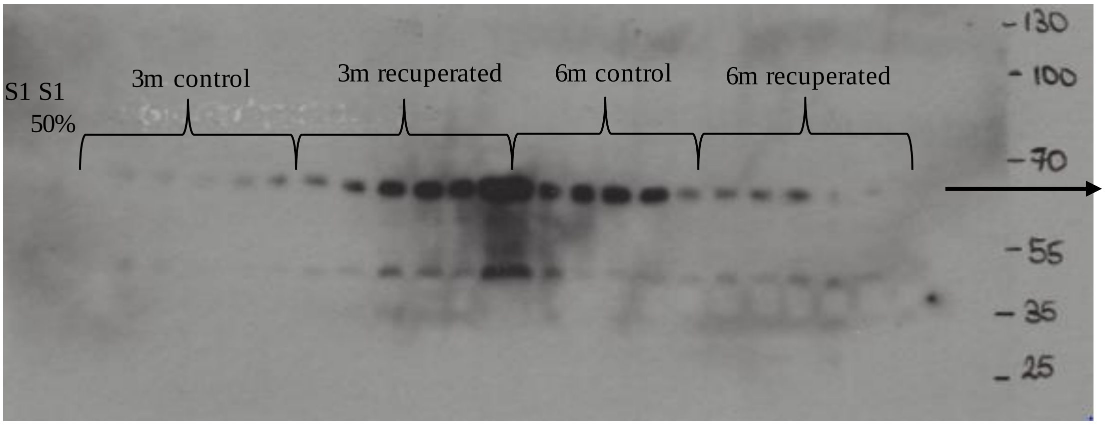

PAkt^{ser473}

Figure 4

a) IL1β (ovarian fat)

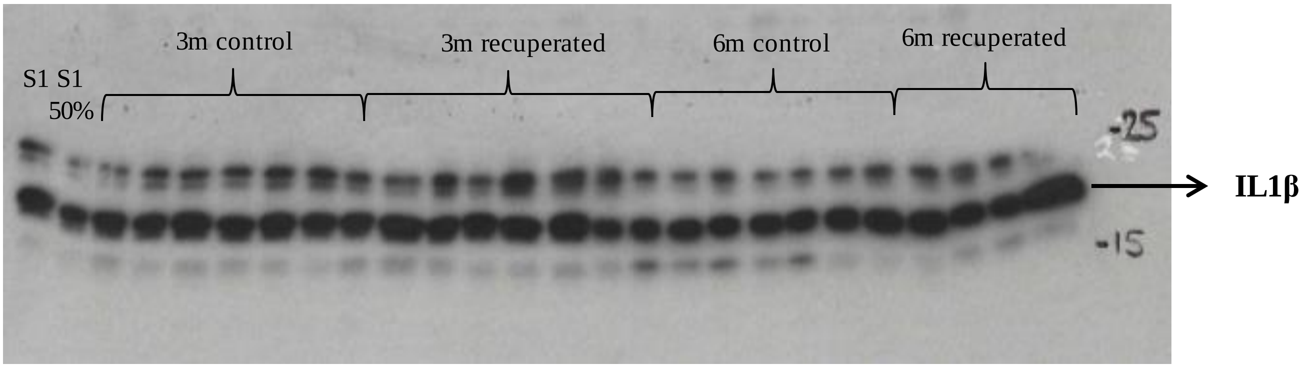

b) IL6 (ovarian fat)

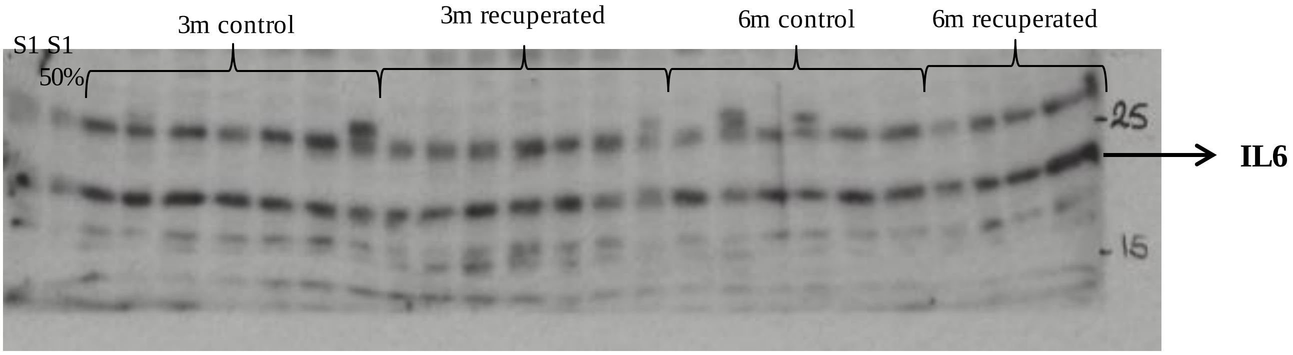

c) Catalase (ovarian fat)

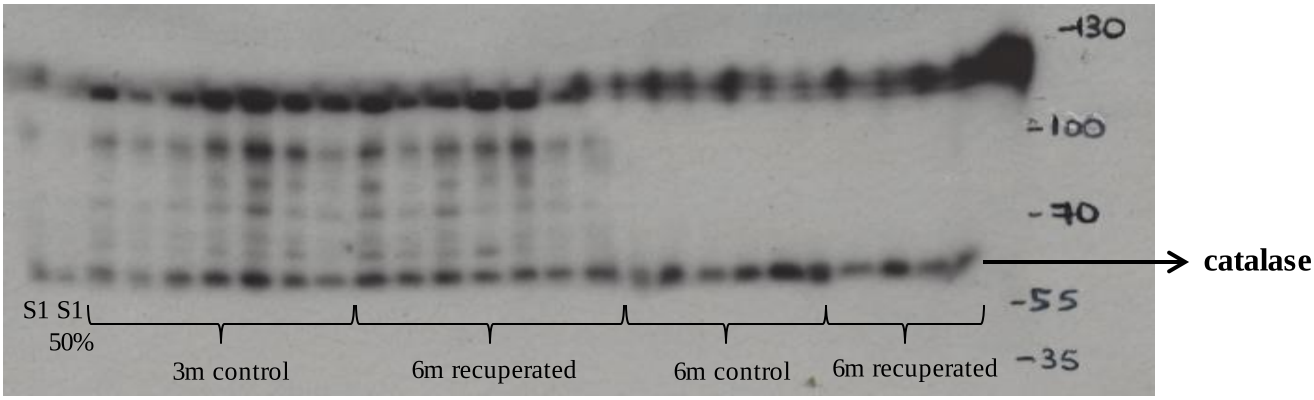

Supplementary Table T1: Control and isocaloric low-protein diet composition

**Component**	**Control (% composition)**	**LP (% composition)**
Vitamin pre-mix	0.25	0.25
St. Spor. Premix	0.25	0.25
$CaHP04.2H_20$	0.65	1.15
CaC03	1.5	1.1
KH2P04	0.7	0.7
KCl	0.7	0.7
Salt	0.3	0.3
MgS04.7H20	0.4	0.4
Mg0 Schwer reinst	0.2	0.2
Methionine Sythn. DL	0.2	0.08
Choline CL 50%	0.4	0.4
Casein protein	22	9.0
Cornstarch gel	8.0	8.0
Dicacel2+4/cellulose	5.0	5.0
Soya oil (fibre)	4.3	4.3
Dextrose	55.15	68.1
Total	100	100

Supplementary data legends

Supplementary Figure 1 S1: The effect of in-utero protein restriction, accelerated postnatal growth and ageing upon overall size distribution of adipocytes in 3 and 6 month female rats a) control 3m vs. control 6m and b) recuperated 3m vs. recuperated 6m. N values = 7 per group.

Supplementary Figure 2 S2: Coommassie blue staining for demonstration of equal protein loading for a) ovarian fat and b) VL skeletal muscle. N values: ovarian fat 3m control =7, 3m recuperated = 7, 6m control = 6, 6m recuperated = 6. VL = 6 per group.

Supplementary Figure S3: Images of full-length Western blots analysed in this study:

Ovarian fat (for data in Figure 2): a) IRβ, c) IRS1, e)PKCζ, f) Akt, g) pAkt^{ser473}, h) P110β. VL skeletal muscle (for data in Figure 3) a) IRβ, b) pIRβY1361, c) Akt and d) pAkt ^{ser473}. Ovarian fat (for data in Figure 4) b) IL1β, d) IL6 and f) Catalase. Note: Due to differing molecular weights of proteins, some proteins were run on the same gel and blots were split according to molecular weights, for protein detection: Ovarian fat: IRS1 and IL6 (exposure time 10 seconds), PKCζ and IL1β (exposure time 5 seconds). pAkt ^{ser473}, Akt and IRβ (exposure time 1 second) and P110β (exposure time 1 minute) were all detected on separate blots. VL skeletal muscle: pIRβ^{Y1361} (exposure time 1 second) and Akt (exposure time 5 minutes) were run on the same blot. IRβ (exposure time 2 minutes) pAkt ^{ser473} (exposure time 5 minutes) were run on separate blots. Optimal exposure times (as seen in images) were determined by the ratio between loading controls (100% and 50% of pooled sample; S1).

Supplementary Table 1 T1: The composition of diets used in this study.
